# Determinants of appropriate antibiotic and NSAID prescribing in unscheduled outpatient settings in the veterans health administration

**DOI:** 10.1186/s12913-024-11082-0

**Published:** 2024-05-18

**Authors:** Michael J. Ward, Michael E. Matheny, Melissa D. Rubenstein, Kemberlee Bonnet, Chloe Dagostino, David G. Schlundt, Shilo Anders, Thomas Reese, Amanda S. Mixon

**Affiliations:** 1 Education, and Clinical Center (GRECC), VA , Geriatric Research, Tennessee Valley Healthcare System, 2525 West End Avenue, Ste. 1430, Nashville, TN 37203 USA; 2 Medicine Service, Tennessee Valley Healthcare System, Nashville, TN USA; 3 Department of Emergency Medicine, Vanderbilt University Medical Center, Nashville, TN USA; 4 Department of Biomedical Informatics, Vanderbilt University Medical Center, Nashville, TN USA; 5 Department of Biostatistics, Vanderbilt University Medical Center, Nashville, TN USA; 6 Division of General Internal Medicine & Public Health, Vanderbilt University Medical Center, Nashville, TN USA; 7 Department of Psychology, Vanderbilt University, Nashville, TN USA; 8 Center for Research and Innovation in Systems Safety, Vanderbilt University Medical Center, Nashville, TN USA; 9 Section of Hospital Medicine, Vanderbilt University Medical Center, Nashville, TN USA

**Keywords:** Non-Steroidal Anti-Inflammatory Drugs, Antibiotics, Qualitative Methods, Emergency Department, Urgent Care, Primary Care, Prescribing Stewardship

## Abstract

**Background:**

Despite efforts to enhance the quality of medication prescribing in outpatient settings, potentially inappropriate prescribing remains common, particularly in unscheduled settings where patients can present with infectious and pain-related complaints. Two of the most commonly prescribed medication classes in outpatient settings with frequent rates of potentially inappropriate prescribing include antibiotics and nonsteroidal anti-inflammatory drugs (NSAIDs). In the setting of persistent inappropriate prescribing, we sought to understand a diverse set of perspectives on the determinants of inappropriate prescribing of antibiotics and NSAIDs in the Veterans Health Administration.

**Methods:**

We conducted a qualitative study guided by the Consolidated Framework for Implementation Research and Theory of Planned Behavior. Semi-structured interviews were conducted with clinicians, stakeholders, and Veterans from March 1, 2021 through December 31, 2021 within the Veteran Affairs Health System in unscheduled outpatient settings at the Tennessee Valley Healthcare System. Stakeholders included clinical operations leadership and methodological experts. Audio-recorded interviews were transcribed and de-identified. Data coding and analysis were conducted by experienced qualitative methodologists adhering to the Consolidated Criteria for Reporting Qualitative Studies guidelines. Analysis was conducted using an iterative inductive/deductive process.

**Results:**

We conducted semi-structured interviews with 66 participants: clinicians (*N* = 25), stakeholders (*N* = 24), and Veterans (*N* = 17). We identified six themes contributing to potentially inappropriate prescribing of antibiotics and NSAIDs: 1) Perceived versus actual Veterans expectations about prescribing; 2) the influence of a time-pressured clinical environment on prescribing stewardship; 3) Limited clinician knowledge, awareness, and willingness to use evidence-based care; 4) Prescriber uncertainties about the Veteran condition at the time of the clinical encounter; 5) Limited communication; and 6) Technology barriers of the electronic health record and patient portal.

**Conclusions:**

The diverse perspectives on prescribing underscore the need for interventions that recognize the detrimental impact of high workload on prescribing stewardship and the need to design interventions with the end-user in mind. This study revealed actionable themes that could be addressed to improve guideline concordant prescribing to enhance the quality of prescribing and to reduce patient harm.

**Supplementary Information:**

The online version contains supplementary material available at 10.1186/s12913-024-11082-0.

## Background

Adverse drug events (ADEs) are the most common iatrogenic injury. [1] Efforts to reduce these events have primarily focused on the inpatient setting. However, the emergency department (ED), urgent care, and urgent primary care clinics are desirable targets for interventions to reduce ADEs because approximately 70% of all outpatient encounters occur in one of these settings. [2] Two of the most commonly prescribed drug classes during acute outpatient care visits that have frequent rates of potentially inappropriate prescribing include antibiotics and non-steroidal anti-inflammatory drugs (NSAIDs). [3, 4]

An estimated 30% of all outpatient oral antibiotic prescriptions may be unnecessary. [5, 6] The World Health Organization identified overuse of antibiotics and its resulting antimicrobial resistance as a global threat. [7] The Centers for Disease Control and Prevention (CDC) conservatively estimates that in the US there are nearly 3 million antibiotic-resistant infections that cause 48,000 deaths annually. [8] Antibiotics were the second most common source of adverse events with nearly one ADE resulting in an ED visit for every 100 prescriptions. [9] Inappropriate antibiotic prescriptions (e.g., antibiotic prescription for a viral infection) also contribute to resistance and iatrogenic infections such as *C. difficile* (antibiotic associated diarrhea) and *Methicillin-resistant Staphylococcus aureus (MRSA)*. [8] NSAID prescriptions, on the other hand, result in an ADE at more than twice the rate of antibiotics (2.2%), [10] are prescribed to patients at an already increased risk of potential ADEs, [4, 11] and frequently interact with other medications. [12] Inappropriate NSAID prescriptions contribute to serious gastrointestinal, [13] renal, [14] and cardiovascular [15, 16] ADEs such as gastrointestinal bleeding, acute kidney injury, and myocardial infarction or heart failure, respectively. Yet, the use of NSAIDs is ubiquitous; according to the CDC, between 2011 and 2014, 5% of the US population were prescribed an NSAID whereas an additional 2% take NSAIDs over the counter. [11]

Interventions to reduce inappropriate antibiotic prescribing commonly take the form of antimicrobial stewardship programs. However, no such national programs exist for NSAIDs, particularly in acute outpatient care settings. There is a substantial body of evidence supporting the evidence of such stewardship programs. [17] The CDC recognizes that such outpatient programs should consist of four core elements of antimicrobial stewardship, [18] including commitment, action for policy and practice, tracking and reporting, and education and expertise. However, the opportunities to extend antimicrobial stewardship in EDs are vast. Despite the effectiveness, there is a recognized need to understand which implementation strategies and how to implement multifaceted interventions. [19] Given the unique time-pressured environment of acute outpatient care settings, not all antimicrobial stewardship strategies work in these settings necessitating the development of approaches tailored to these environments. [19, 20]

One particularly vulnerable population is within the Veterans Health Administration. With more than 9 million enrollees in the Veterans Health Administration, Veterans who receive care in Veteran Affairs (VA) hospitals and outpatient clinics may be particularly vulnerable to ADEs. Older Veterans have greater medical needs than younger patients, given their concomitant medical and mental health conditions as well as cognitive and social issues. Among Veterans seen in VA EDs and Urgent Care Clinics (UCCs), 50% are age 65 and older, [21] nearly three times the rate of non-VA emergency care settings (18%). [22] Inappropriate prescribing in ED and UCC settings is problematic with inappropriate antibiotic prescribing estimated to be higher than 40%. [23] In a sample of older Veterans discharged from VA ED and UCC settings, NSAIDs were found to be implicated in 77% of drug interactions. [24]

Learning from antimicrobial stewardship programs and applying to a broader base of prescribing in acute outpatient care settings, it is necessary to understand not only why potentially inappropriate prescribing remains a problem for antibiotics, but for medications (e.g., NSAIDs) which have received little stewardship focus previously. This understanding is essential to develop and implement interventions to reduce iatrogenic harm for vulnerable patients seen in unscheduled settings. In the setting of the Veterans Health Administration, we sought to use these two drug classes (antibiotics and NSAIDs) that have frequent rates of inappropriate prescribing in unscheduled outpatient care settings, to understand a diverse set of perspectives on why potentially inappropriate prescribing continues to occur.

## Methods

### Selection of participants

Participants were recruited from three groups in outpatient settings representing emergency care, urgent care, and urgent primary care in the VA: 1) Clinicians-VA clinicians such as physicians, advanced practice providers, and pharmacists 2) Stakeholders-VA and non-VA clinical operational and clinical content experts such as local and regional medical directors, national clinical, research, and administrative leadership in emergency care, primary care, and pharmacy including geriatrics; and 3) Veterans seeking unscheduled care for infectious or pain symptoms.

Clinicians and stakeholders were recruited using email, informational flyers, faculty/staff meetings, national conferences, and snowball sampling, when existing participants identify additional potential research subjects for recruitment. [25] Snowball sampling is useful for identifying and recruiting participants who may not be readily apparent to investigators and/or hard to reach. Clinician inclusion criteria consisted of: 1) at least 1 year of VA experience; and 2) ≥ 1 clinical shift in the last 30 days at any VA ED, urgent care, or primary care setting in which unscheduled visits occur. Veterans were recruited in-person at the VA by key study personnel. Inclusion criteria consisted of: 1) clinically stable as determined by the treating clinician; 2) 18 years or older; and 3) seeking care for infectious or pain symptoms in the local VA Tennessee Valley Healthcare System (TVHS). TVHS includes an ED at the Nashville campus with over 30,000 annual visits, urgent care clinic in Murfreesboro, TN with approximately 15,000 annual visits, and multiple primary care locations throughout the middle Tennessee region. This study was approved by the VA TVHS Institutional Review Board as minimal risk.

### Data collection

Semi-structured interview guides (Supplemental Table 1) were developed using the Consolidated Framework for Implementation Research (CFIR) [26] and the Theory of Planned Behavior [27, 28] to understand attitudes and beliefs as they relate to behaviors, and potential determinants of a future intervention. Interview guides were modified and finalized by conducting pilot interviews with three members of each participant group. Interview guides were tailored to each group of respondents and consisted of questions relating to: 1) determinants of potentially inappropriate prescribing; and 2) integration into practice (Table. 1). Clinicians were also asked about knowledge and awareness of evidence-based prescribing practices for antibiotics and NSAIDs. The interviewer asked follow-up questions to elicit clarity of responses and detail.

**Table 1:** Summaries of Key Interview Guide Questions for A Clinicians, B Stakeholders, and C Veterans

**A. Clinician Interview Guide Summarized Questions** 1) Determinants of Potentially Inappropriate Medication Prescriptions · Describe antibiotic prescribing at your site. · What types of situations are there in which antibiotics or NSAIDs might not be prescribed? · Describe challenges communicating with patients about antibiotics and NSAIDs. 2) Knowledge and Awareness · Describe your decision-making process for prescription decisions. · At your site, do you think that antibiotics or NSAIDs are prescribed when maybe they shouldn’t be? · Do you ever hear about adverse events when these medications are prescribed? · What existing efforts outside of your control influence how you prescribe antibiotics and NSAIDs? · If an antibiotic or NSAID caused harm to one of your or your colleagues’ patients, how would this influence your prescribing? · There are several interventions to facilitate change, which would you find most helpful? Which of the following interventions would be most helpful to facilitate change; Learning about your own prescribing patterns, Specific patient adverse events, Real-time clinical decision support, Peer expert, Incentives to follow-up on patients. 3) Integration into Practice · Discuss how you would interact with the data provided in the feedback report. · Describe a desirable conversation about your prescribing with a pharmacist.
**B. Stakeholder Interview Guide Summarized Questions** 1) Determinants of Potentially Inappropriate Prescribing · What types of situations are there in which antibiotics or NSAIDs might not be prescribed? · Describe your experience with prescribing in acute care settings. · Discuss the main barriers in addressing potentially inappropriate prescribing? · Describe possible strategies to address this problem. 2) Integration into Practice · Describe what clinicians should do with these data. · Discuss your experience with any existing similar reports. · Do you think the use of non-financial incentives would motivate a clinician to review the report? · Describe how this intervention should be implemented in clinical practice on a nation scale. · Describe the potential barriers and facilitators to implementing this intervention on a broader scale.
**C. Veteran Interview Guide Summarized Questions ** 1) Determinants of Potentially Inappropriate Medication Prescriptions · Do you expect to leave with a prescription today? · What information about a prescribed medication would you like to know? 2) Integration into Practice · Discuss what you would want to know about an antibiotic prescription. · Discuss what you would want to know about a NSAID prescription. · Discuss what you would want to know if not prescribed a medication. · Describe the desired discussion with your clinician about their prescription decision. · Describe education materials that would be helpful to understand the clinician’s prescription decision.

Each interview was conducted by a trained interviewer (MDR). Veteran interviews were conducted in-person while Veterans waited for clinical care so as not to disrupt clinical operations. Interviews with clinicians and stakeholders were scheduled virtually. All interviews (including in-person) were recorded and transcribed in a manner compliant with VA information security policies using Microsoft Teams (Redmond, WA). The audio-recorded interviews were transcribed and de-identified by a transcriptionist and stored securely behind the VA firewall using Microsoft Teams. Study personnel maintained a recording log on a password-protected server and each participant was assigned a unique participant ID number. Once 15 interviews were conducted per group, we planned to review interviews with the study team to discuss content, findings, and to decide collectively when thematic saturation was achieved, the point at which no new information was obtained. [29] If not achieved, we planned to conduct at least 2 additional interviews prior to group review for saturation. We estimated that approximately 20–25 interviews per group were needed to achieve thematic saturation.

### Analysis

Qualitative data coding and analysis was managed by the Vanderbilt University Qualitative Research Core. A hierarchical coding system (Supplemental Table 2) was developed and refined using an iterative inductive/deductive approach [30–32] guided by a combination of: 1) Consolidated Framework for Implementation Research (CFIR) [26]; 2) the Theory of Planned Behavior [27, 28]; 3) interview guide questions; and 4) a preliminary review of the transcripts. Eighteen major categories (Supplemental Table 3) were identified and were further divided into subcategories, with some subcategories having additional levels of hierarchical division. Definitions and rules were written for the use of each of the coding categories. The process was iterative in that the coding system was both theoretically informed and derived from the qualitative data. The coding system was finalized after it was piloted by the coders. Data coding and analysis met the Consolidated Criteria for Reporting Qualitative Research (COREQ) guidelines. [33]

Four experienced qualitative coders were trained by independently coding two transcripts from each of the three participant categories. Coding was then compared, and any discrepancies resolved by reconciliation. After establishing reliability in using the coding system, the coders divided and independently coded the remaining transcripts in sequential order. Each statement was treated as a separate quote and could be assigned up to 21 different codes. Coded transcripts were combined and sorted by code.

Following thematic saturation, the frequency of each code was calculated to understand the distribution of quotes. Quotes were then cross-referenced with coding as a barrier to understand potential determinants of inappropriate prescribing. A thematic analysis of the barriers was conducted and presented in an iterative process with the research team of qualitative methodologists and clinicians to understand the nuances and refine the themes and subthemes from the coded transcripts. Transcripts, quotations, and codes were managed using Microsoft Excel and SPSS version 28.0.

## Results

We approached 132 individuals and 66 (50%) agreed to be interviewed. Participants included 25 clinicians, 24 stakeholders, and 17 Veterans whose demographic characteristics are presented in Table 2. The clinicians were from 14 VA facilities throughout the US and 20 physicians, and five advanced practice providers. Of the clinicians, 21 (84%) worked in either an ED or urgent care while the remainder practiced in primary care. The 24 stakeholders included 13 (54%) clinical service chiefs or deputy chief (including medical directors), five (21%) national directors, and six (25%) experts in clinical content and methodology. The 17 Veterans interviewed included 15 (88%) who were seen for pain complaints.

**Table 2:** Participant demographics (*N* = 66)

	**Clinicians (** ***N*** ** = 25)**	**Stakeholders (** ***N*** ** = 24)**	**Veterans (** ***N*** ** = 17)**	
Median Age, years (IQR)	43 years (36, 59.5)	48 years (45.25, 54)	64 years (51, 72.5)	
Female Sex, N (%)	16 (64%)	10 (42%)	2 (15%)	
Years Experience	11, (7, 28.5)	17.5 (5.5, 20.5)	-	
Role	APP 5 (20%) Physician 20 (80%)	Clinical Service Chief^a^ 13 National Directors 5 Clinical Content Expert 6	-	
Setting	ED/Urgent Care 21 (84%) Primary Care 4 (16%)	-	ED 11(65%) Urgent Care 6 (35%)	
Reason for Visit	-	-	Pain 15 (88%) Infection 2 (12%)	

*APP* advanced practice provider, *ED* emergency department, *IQR* interquartile range

^a^Includes Deputy chiefs and medical director roles

**Fig. 1:**
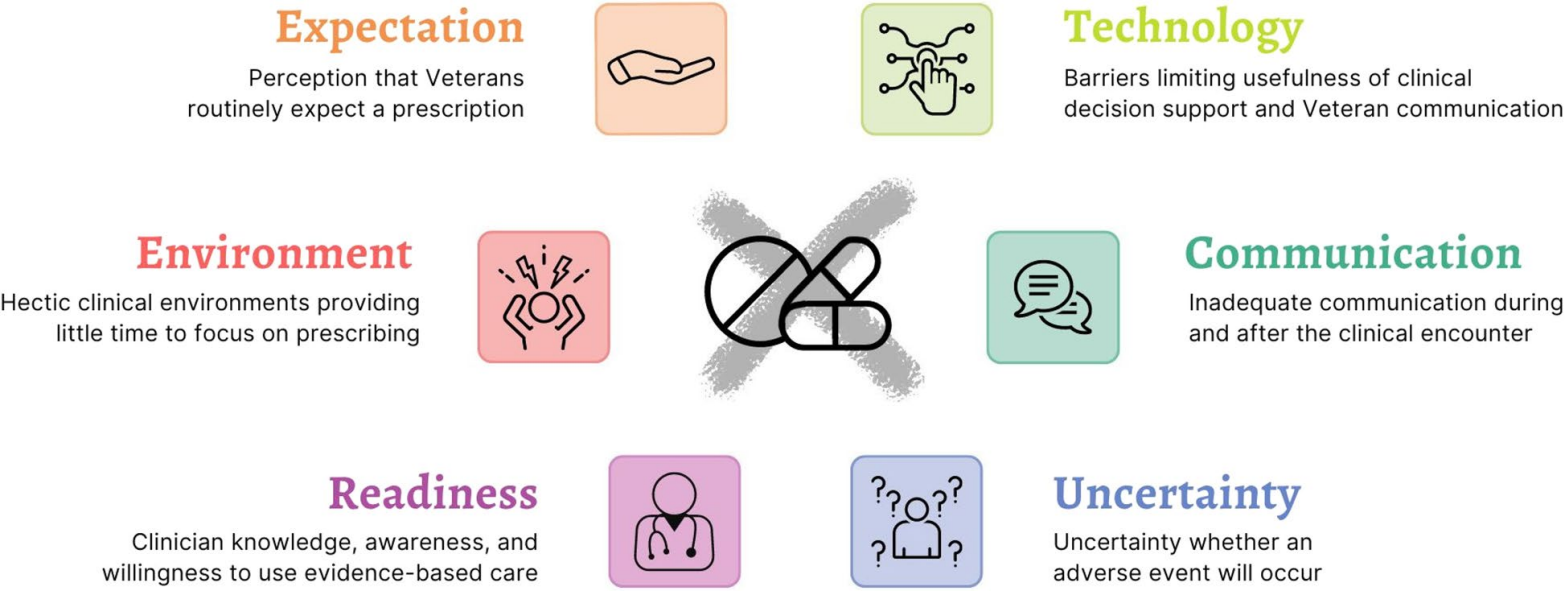
Visual representation of themes and subthemes from 66 clinician, stakeholder, and Veteran interviews

Results are organized by the six thematic categories with several subthemes in each category. Themes and subthemes are presented in Table 3 and are visually represented in Fig. 1. The six themes were: 1) perceived versus actual Veterans expectations about prescribing, 2) the influence of a time-pressured clinical environment on prescribing stewardship, 3) limited clinician knowledge, awareness, and willingness to use evidence-based care, 4) uncertainties about the Veteran condition at the time of the clinical encounter, 5) limited communication, and 6) technology barriers.

**Table 3:** Themes and Subthemes from Interviews

Theme 1: Perception that Veterans routinely expect a medication from their visit, despite clinical appropriateness There is a pressure to “do something” that frequently involves providing a prescription and may be contrary to clinical appropriateness Potential outside influences that contribute to medication expectation Workload and patient satisfaction may suffer if a prescription is not provided Veterans do not expect a medication, they want to get better
Theme 2: A frequently hectic clinical environment and unique practice conditions in unscheduled settings provided little time to focus on prescribing practices Time pressured environment provides little time to focus on prescribing Unique practice conditions making a clinician’s patients “different” Practice norms impact prescribing behavior
Theme 3: Clinician knowledge, awareness, and willingness to use evidence-based care Lack of clinician awareness of potential comorbidities and drug interactions Clinician willingness to change behavior
Theme 4: Uncertainty about whether an adverse event will occur Challenges in knowing whether a Veteran’s condition would be appropriate for an NSAID Prescribing antibiotics “out of fear” to prevent adverse events
Theme 5: Inadequate communication during and after the clinical encounter Limited communication with primary care Lack of post-encounter feedback Veteran communication preferences during the clinical encounter about medication information Lack of Veteran interest in handouts, posters, and web sites
Theme 6: Technology barriers limited the usefulness of clinical decision support and patient communication Electronic health record pop-up fatigue Challenges access the Veteran patient portal, MyHealtheVet

### Theme 1: Perception that Veterans routinely expect a medication from their visit, despite clinical inappropriateness

According to clinicians, Veterans frequently expect to receive a prescription even when this decision conflicts with good clinical practice.

*Certainly lots of people would say you know if you feel like you’re up against some strong expectations from the patients or caregivers or families around the utility of an antibiotic when it’s probably not indicated…In the emergency department the bias is to act and assume the worst and assume like the worst for the clinical trajectory for the patient rather than the reverse.* [Clinician 49, Physician, ED]

In addition, stakeholders further stated that patient prescription expectations are quite influential and are likely shaped by Veterans’ prior experiences.

*I think the patients, particularly for antibiotics, have strong feelings about whether they should or shouldn’t get something prescribed.* [Stakeholder 34]

*You know I think the biggest challenge, I think, is adjusting patients’ expectations because you know they got better the last time they were doing an antibiotic.* [Stakeholder 64]

Patient satisfaction and clinician workload may also influence the clinician’s prescription decision.

*We have a lot of patients that come in with back pain or knee pain or something. We’ll get an x-ray and see there’s nothing actually wrong physically that can be identified on x-ray at least and you have to do something. Otherwise, patient satisfaction will dip, and patients leave angry.* [Clinician 28, Physician, urgent care clinic]

*For some clinicians it’s just easier to prescribe an antibiotic when they know that’s the patient’s expectation and it shortens their in-room discussion and evaluation.* [Clinician 55, Physician, ED]

Despite clinician perception, Veterans communicated that they did not necessarily expect a prescription and were instead focused on the clinical interaction and the clinician’s decision.

*I’m not sure if they’ll give me [unintelligible] a prescription or what they’ll do. I don’t care as long as they stop the pain.* [Patient 40, urgent care clinic]

*I don’t expect to [receive a prescription], but I mean whatever the doctor finds is wrong with me I will follow what he says.* [Patient 31, ED]

### Theme 2: Hectic clinical environments and unique practice conditions in unscheduled settings provide little time to focus on prescribing practices

Clinicians and stakeholders reported that the time-constrained clinical environment and need to move onto the next patient were major challenges to prescribing stewardship.

*The number one reason is to get a patient out of your office or exam bay and move on to the next one.* [Stakeholder 28]

*It takes a lot of time and you have to be very patient and understanding. So, you end up having to put a fair bit of emotional investment and intelligence into an encounter to not prescribe.* [Stakeholder 1]

Stakeholders also noted that unique shift conditions and clinician perceptions that their patients were “different” might influence prescribing practices.

*A common pushback was ‘well my patients are different.’* [Stakeholder 4]

*Providers who worked different types of shifts, so if you happened to work on a Monday when the clinics were open and had more adults from the clinics you were more likely to prescribe antibiotics than if you worked over night and had fewer patients. Providers who worked primarily holidays or your Friday prescribing pattern may be very different if you could get them into a primary care provider the next day.* [Stakeholder 22]

Clinicians also reported that historical practices in the clinical environment practices may also contribute to inappropriate prescribing.

*I came from working in the [outpatient] Clinic as a new grad and they’re very strict about prescribing only according to evidence-based practice. And then when I came here things are with other colleagues are a little more loose with that type of thing. It can be difficult because you start to adopt that practice to.* [Clinician 61, Nurse Practitioner, ED]

### Theme 3: Clinician knowledge, awareness, and willingness to use evidence-based care

Stakeholders felt that clinicians had a lack of knowledge about prescribing of NSAIDs and antibiotics.

*Sometimes errors are a lack of knowledge or awareness of the need to maybe specifically dose for let’s say impaired kidney function or awareness of current up to date current antibiotic resistance patterns in the location that might inform a more tailored antibiotic choice for a given condition.* [Stakeholder 37]

*NSAIDs are very commonly used in the emergency department for patients of all ages…the ED clinician is simply not being aware that for specific populations this is not recommended and again just doing routine practice for patients of all ages and not realizing that for older patients you actually probably should not be using NSAIDs.* [Stakeholder 40]

Some clinicians may be unwilling to change their prescribing practices due to outright resistance, entrenched habits, or lack of interest in doing so.

*It sounds silly but there’s always some opposition to people being mandated to do something. But there are some people who would look and go ‘okay we already have a handle on that so why do we need something else? I know who prescribes inappropriately and who doesn’t. Is this a requirement, am I evaluated on it? That would come from supervisors. Is this one more thing on my annual review?’* [Stakeholder 28]

*If people have entrenched habits that are difficult to change and are physicians are very individualistic people who think that they are right more often than the non-physician because of their expensive training and perception of professionalism.* [Stakeholder 4]

### Theme 4: Uncertainty about whether an adverse event will occur

Clinicians cited the challenge of understanding the entirety of a Veteran’s condition, potential drug-drug interactions, and existing comorbidities in knowing whether an NSAID prescription may result in an adverse event.

*It’s oftentimes a judgement call if someone has renal function that’s right at the precipice of being too poor to merit getting NSAIDs that may potentially cause issues.* [Clinician 43, Physician, inpatient and urgent care]

*It depends on what the harm is. So, for instance, you can’t always predict allergic reactions. Harm from the non-steroidals would be more if you didn’t pre-identify risk factors for harm. So, they have ulcer disease, they have kidney problems where a non-steroidal would not be appropriate for that patient. Or potential for a drug-drug interaction between that non-steroid and another medication in particular.* [Clinician 16, Physician, ED]

Rather than be concerned about the adverse events resulting from the medication itself, stakeholders identified the uncertainty that clinicians experience about whether a Veteran may experience an adverse event from an infection if nothing is done. This uncertainty contributes to the prescription of an antibiotic.

*My experience in working with providers at the VA over the years is that they worry more about the consequences of not treating an infection than about the consequences of the antibiotic itself.* [Stakeholder 19]

*Sometimes folks like to practice conservatively and they’ll say even though I didn’t really see any hard evidence of a bacterial infection, the patient’s older and sicker and they didn’t want to risk it.* [Stakeholder 16]

### Theme 5: Limited communication during and after the clinical encounter

The role and type of communication about prescribing depended upon the respondent. Clinicians identified inadequate communication and coordination with the Veteran’s primary care physician during the clinical encounter.

*I would like to have a little more communication with the primary doctors. They don’t seem to be super interested in talking to anyone in the emergency room about their patients… A lot of times you don’t get an answer from the primary doctor or you get I’m busy in clinic. You can just pick something or just do what you think is right.* [Clinician 25, Physician, ED]

Alternatively, stakeholders identified post-encounter patient outcome and clinical performance feedback as potential barriers.

*Physicians tend to think that they are doing their best for every individual patient and without getting patient by patient feedback there is a strong cognitive bias to think well there must have been some exception and reason that I did it in this setting.* [Stakeholder 34]

*It’s really more their own awareness of like their clinical performance and how they’re doing.* [Stakeholder 40]

Veterans, however, prioritized communication during the clinical encounter. They expressed the need for clear and informative communication with the clinician, and the need for the clinician to provide a rationale for the choice and medication-specific details along with a need to ask any questions.

*I expect him to tell me why I’m taking it, what it should do, and probably the side effects.* [Patient 25, ED]

*I’d like to have a better description of how to take it because I won’t remember all the time and sometimes what they put on the bottle is not quite as clear.* [Patient 22, ED]

Veterans reported their desire for a simple way to learn about medication information. They provided feedback on the current approaches to educational materials about prescriptions.

*Probably most pamphlets that people get they’re not going to pay attention to them. Websites can be overwhelming.* [Patient 3, ED]

*Posters can be offsetting. If you’re sick, you’re not going to read them…if you’re sick you may glance at that poster and disregard it. So, you’re not really going to see it but if you give them something in the hand people will tend to look at it because it’s in their hand.* [Patient 19, ED]

*It would be nice if labels or something just told me what I needed to know. You know take this exactly when and reminds me here’s why you’re taking it for and just real clear and not small letters.* [Patient 7, ED]

### Theme 6: Technology barriers limited the usefulness of clinical decision support for order checking and patient communication tools

Following the decision to prescribe a medication, clinicians complained that electronic health record pop-ups with clinical decision support warnings for potential safety concerns (e.g., drug-drug interactions) were both excessive and not useful in a busy clinical environment.

*The more the pop ups, the more they get ignored. So, it’s finding that sweet spot right where you’re not constantly having to click out of something because you’re so busy. Particularly in our clinical setting where we have very limited amount of time to read the little monograph. Most of the time you click ‘no’ and off you go.* (Clinician 16, Physician, ED)

*Some of these mechanisms like the EMR [electronic medical record] or pop-up decision-making windows really limit your time. If you know the guidelines appropriately and doing the right thing, even if you’re doing the right thing it takes you a long time to get through something.* (Clinician 19, Physician, Primary care clinic)

For post-encounter communication that builds on Theme 5 about patient communication, patients reported finding using the VA patient portal (MyHealtheVet) challenging for post-event communication with their primary care physician and to review the medications they were prescribed.

*I’ve got to get help to get onto MyHealtheVet but I would probably like to try and use that, but I haven’t been on it in quite some time.* [Patient 22, ED]

*I tried it [MyHealtheVet] once and it’s just too complicated so I’m not going to deal with it.* [Patient 37, Urgent care]

## Discussion

This work examined attitudes and perceptions of barriers to appropriate prescribing of antibiotics and NSAIDs in unscheduled outpatient care settings in the Veterans Health Administration. Expanding on prior qualitative work on antimicrobial stewardship programs, we also included an examination of NSAID prescribing, a medication class which has received little attention focused on prescribing stewardship. This work seeks to advance the understanding of fundamental problems underlying prescribing stewardship to facilitate interventions designed to improve not only the decision to prescribe antibiotics and NSAIDs, but enhances the safety checks once a decision to prescribe is made. Specifically, we identified six themes during these interviews: perceived versus actual Veteran expectations about prescribing, the influence of a time-pressured clinical environment on prescribing stewardship, limited clinician knowledge, awareness, and willingness to use evidence-based care, uncertainties about the Veteran condition at the time of the clinical encounter, limited communication, and technology barriers.

Sensitive to patient expectations, clinicians believed that Veterans would be dissatisfied if they did not receive an antibiotic prescription, [34] even though most patients presenting to the ED for upper respiratory tract infections do not expect antibiotics. [35] However, recent work by Staub et al. found that among patients with respiratory tract infections, receipt of an antibiotic was not independently associated with improved satisfaction. [36] Instead, they found that receipt of antibiotics had to match the patient’s expectations to affect patient satisfaction and recommended that clinicians communicate with their patients about prescribing expectations. This finding complements our results in the present study and the importance of communication about expectations is similarly important for NSAID prescribing as well.

A commitment to stewardship and modification of clinician behavior may be compromised by the time-pressured clinical environment, numerous potential drug interactions, comorbidities of a vulnerable Veteran population, and normative practices. The decision to prescribe medications such as antibiotics is a complex clinical decision and may be influenced by both clinical and non-clinical factors. [34, 37, 38] ED crowding, which occurs when the demand for services exceeds a system’s ability to provide care, [39] is a well-recognized manifestation of a chaotic clinical environment and is associated with detrimental effects on the hospital system and patient outcomes. [40, 41] The likelihood that congestion and wait times will improve is unlikely as the COVID-19 pandemic has exacerbated the already existing crowding and boarding crisis in EDs. [42, 43]

Another theme was the uncertainty in the anticipation of adverse events that was exacerbated by the lack of a feedback loop. Feedback on clinical care processes and patient outcomes is uncommonly provided in emergency care settings, [44] yet may provide an opportunity to change clinician behavior, particularly for antimicrobial stewardship. [45] However, the frequent use of ineffective feedback strategies [46] compromises the ability to implement effective feedback interventions; feedback must be specific [47] and address the Intention-to-Action gap [48] by including co-interventions to address recipient characteristics (i.e., beliefs and capabilities) and context to maximize impact. Without these, feedback may be ineffective.

An additional barrier identified from this work is the limited communication with primary care following discharge. A 2017 National Quality Forum report on ED care transitions [49] recommended that EDs and their supporting hospital systems should expand infrastructure and enhance health information technology to support care transitions as Veterans may not understand discharge instructions, may not receive post-ED or urgent care, [50–52] or may not receive a newly prescribed medication. [24] While there are existing mechanisms to communicate between the ED and primary care teams such as notifications when a Veteran presents to the ED and when an emergency clinician copies a primary care physician on a note, these mechanisms are insufficient to address care transition gaps and are variable in best practice use. To address this variability, the VA ED PACT Tool was developed using best practices (standardized processes, "closed-loop" communication, embedding into workflow) to facilitate and standardize communication between VA EDs and follow-up care clinicians. [53] While the ED PACT Tool is implemented at the Greater Los Angeles VA and can create a care coordination order upon ED discharge, its use is not yet widely adopted throughout the VA.

In the final theme about technology barriers, once the decision has been made to prescribe a medication, existing electronic tools that are key components of existing stewardship interventions designed to curtail potentially inappropriate prescriptions may be compromised by their lack of usability. For example, clinician and stakeholder interview respondents described how usability concerns were exacerbated in a time-pressured clinical environment (e.g., electronic health record clinical decision support tools). Clinical decision support is an effective tool to improve healthcare process measures in a diverse group of clinical environments; [54] however, usability remains a barrier when alerts must be frequently overridden. [55, 56] Alert fatigue, as expressed in our interviews for order checking and recognized within the VA’s EHR, [57, 58] may contribute to excessive overrides reducing the benefit of clinical decision support, [56, 59] there was a notable lack of discussion about the decision to initiate appropriate prescriptions, which is a key action of the CDC’s outpatient antibiotic stewardship campaign. [18] Thus, a potentially more effective, albeit challenging approach, is to “nudge” clinicians towards appropriate prescribing and away from the initial decision to prescribe (e.g., inappropriate antibiotic prescribing for viral upper respiratory tract infections) with either default order sets for symptom management or to enhance prescription decisions through reminders about potential contraindications to specific indications (e.g., high risk comorbidities). Beyond EHR-based solutions that might change clinician behavior, the CDC’s outpatient antibiotic stewardship program provides a framework to change the normative practices around inappropriate prescribing and includes a commitment to appropriate prescribing, action for policy and change, tracking and reporting, and education and expertise. [18]

Another technical barrier faces patients through patient-facing electronic tools such as the VA’s MyHealtheVet portal, which was developed to enhance patient communication following care transitions and to allow Veterans to review their medications and to communicate with their primary care clinical team. Patient portals can be an effective tool for medication adherence [60] and offer promise to provide patient education [61] following a clinical encounter. However, they are similarly limited by usability concerns, representing an adoption barrier to broader Veteran use after unscheduled outpatient care visits [62], particularly in an older patient population.

These interviews further underscored that lack of usability of clinical decision support for order checking that arises from ineffective design and is a key barrier preventing health information technology from reaching its promise of improving patient safety. [63] A common and recognized reason for these design challenges include the failure to place the user (i.e., acute care clinician) at the center of the design process resulting in underutilization, workarounds, [64] and unintended consequences, [65] all of which diminish patient safety practices and fail to change clinician behavior (i.e., prescribing). Complex adaptive systems work best when the relative strengths of humans (e.g., context sensitivity, situation specificity) are properly integrated with the information processing power of computerized systems. [66] One potential approach to address usability concerns is through the integration of user-centered design into technology design represents an opportunity to design more clinician- and patient-centric systems of care to advance prescribing stewardship interventions that may have lacked broader adoption previously. As antimicrobial stewardship and additional prescribing stewardship efforts focus on time-pressured environments where usability is essential to adoption, taking a user-centered design approach to not only the development of electronic tools but also in addressing the identified barriers in prescribing represents a promising approach to enhance the quality of prescribing.

### Limitations

The study findings should be considered in light of its limitations. First, the setting for this work was the Veterans Health Administration, the largest integrated health system in the US. Also, while we focused on the stewardship of two drug classes, there are numerous additional drug classes that are prescribed in these settings. Studies in other settings or on other drug classes may not generalize to other settings and drug classes. Second, while clinicians and stakeholder perspectives included diverse, national representation, the Veterans interviewed were local to the Tennessee Valley Healthcare System. Given the concurrent COVID-19 pandemic at the time of enrollment, most of the Veterans were seen for pain-related complaints, and only two infectious-related complaints were included. However, we also asked them about antibiotic prescribing. Clinician and stakeholder narratives may not completely reflect their practice patterns as their responses could be influenced by social desirability bias. Third, responses may be subject to recall bias and may influence the data collected. Finally, the themes and subthemes identified may overlap and have potential interactions. While we used an iterative process to identify discrete themes and subthemes, prescription decisions represent a complex decision process that are influenced by numerous patient and contextual factors and may not be completely independent.

## Conclusions

Despite numerous interventions to improve the quality of prescribing, the appropriate prescription of antibiotics and NSAIDs in unscheduled outpatient care settings remains a challenge. Using the Veterans Health Administration, this study found that challenges to high quality prescribing include perceived Veteran expectations about receipt of medications, a hectic clinical environment deprioritizing stewardship, limited clinician knowledge, awareness, and willingness to use evidence-based care, uncertainty about the potential for adverse events, limited communication, and technology barriers. Findings from these interviews suggest that interventions should consider the detrimental impact of high workload on prescribing stewardship, clinician workflow, the initial decision to prescribe medications, and incorporate end-users into the intervention design process. Doing so is a promising approach to enhance adoption of high quality prescribing practices in order to improve the quality and patient outcomes from NSAID and antibiotic prescribing.

### Supplementary Information

Supplementary Material 1.

## Data Availability

De-identified datasets used and/or analysed during the current study will be made available from the corresponding author on reasonable request.
