# Supplementary material for: Determinants of appropriate antibiotic and NSAID prescribing in unscheduled outpatient settings in the veterans health administration
Source: BMC Health Serv Res. 2024 May 18;24:640. doi: 10.1186/s12913-024-11082-0 (PMC11102113; doi:10.1186/s12913-024-11082-0)
Supplement: Supplementary file 1 — Supplementary Material 1. [file 12913_2024_11082_MOESM1_ESM.pdf]

SUPPLEMENTAL MATERIAL

SUPPLEMENTAL TABLE 1. Interview Guides (Veteran/Family, Provider, Stakeholder)

SUPPLEMENTAL TABLE 2. Coding Framework

SUPPLEMENTAL TABLE 3. Major Coding Categories for Qualitative Analysis

SUPPLEMENTAL TABLE 1. INTERVIEW GUIDES

A. Veteran/Family Interview Guide

Introductory Questions (The following directed at the patient, but can be re-oriented to a witnessing family member)

What we would like to do is better understand how to communicate about prescriptions given to patients in settings like the emergency department, urgent care, and primary care so that we can improve the quality and safety of these processes. So, thank you so much for helping us. While we are talking about prescriptions please know that this **will not have an impact on your care today**, I am asking you these questions so that we can create better communication between physicians and patients when it comes to prescribing medications.

1. What brought you to the clinic today?
    a. Why is this the place you chose to come? (be sure to unpack the answer as much as possible- e.g., if they say that they have had good experiences at this location, follow up with "will you give me some examples of those past experiences?")

The following questions are about medications prescribed by your medical provider. And again, our conversation about prescriptions will not have an impact on your care today.

I. Determinants of Potentially Inappropriate Medication Prescriptions

1. Do you expect to leave with a prescription today?
    a. Why?
    b. What type? Why this type?
2. When your doctor prescribes a medication, what information do you expect them to tell you about the medicine?
    a. Potential side effects or complications?

II. Integration into Practice

1. Antibiotics are used to treat infections caused by bacteria. If your doctor prescribes an antibiotic, what would you want to know about the medicine?
    a. What would you consider a valid reason (ex. Side effects (what kind, pain vs bleeding), drug interactions?)
    b. Alternative treatment- (e.g., if expecting antibiotic: If not an antibiotic what would be an alternative you would be comfortable with?
    c. What else would you want to know?
2. Pain relievers and fever reducers like Aspirin or ibuprofen are known as "NSAIDS", nonsteroidal anti-inflammatory drugs, If your doctor prescribes an NSAID what would you want to know about the medicine?
    a. What would you consider a valid reason (ex. Side effects (what kind, pain vs bleeding), drug interactions?)
    b. Alternative treatment- (e.g., if expecting antibiotic: If not an antibiotic what would be an alternative you would be comfortable with?
    c. What else would you want to know?
3. If your doctor decides not to give you a medication, what questions would you have? I'm not saying that will happen, but what questions would you have?
    a. What would you consider a valid reason (ex. Side effects (what kind, pain vs bleeding), drug interactions?)

    b. Alternative treatment- (e.g., if expecting antibiotic: If not an antibiotic what would be an alternative you would be comfortable with?
    c. What else would you want to know?
4. How would you want the doctor to discuss their prescription decision with you?
    a. (Tailor to antibiotic or NSAID) Given that the time you spend with the doctor might be limited at what point in your visit would you want the doctor to explain why they will not be prescribing a prescription?
    b. Timing of discussion (during vs. end of visit)? (would you want to know up front you would not be prescribed an antibiotic than at the end)
    c. How does this conversation need to go? Non-medical terms? Vs Medical Terminology?
5. What educational materials would be useful to help you to better understand the doctor's decision about whether to prescribe an antibiotic?
    a. Handouts/ pamphlets
    b. Websites
    c. Posters in the room
    d. MyHealththeVet message portal

Conclusion

1. Are there any other thoughts you have about how your doctor prescribes medicine that we did not talk about? Again, we are trying to understand how to communicate about prescriptions given to patients- anything else you can share to help us?

B. <u>Provider Interview Guide</u>

Introductory Questions

1. Please describe your general responsibilities.
2. How often do you see patients for infection?
3. How often do you see patients for pain?
4. How do you hear about your patients after you care for them in your clinical setting?
    a. What do you typically hear?

Key Questions

The next set of questions we are going to discuss involve the prescription of antibiotics and/or non-steroidal anti-inflammatory drugs (NSAIDs) in your clinical setting:

I. <u>Determinants of Potentially Inappropriate Medication Prescriptions</u> (by determinant)

1. So just to start off, will you talk to me about how much of a problem antibiotic prescribing is at your site? Explain
    a. From what I'm learning sometimes providers recognize that there may be reasons not to prescribe antibiotics, but they go ahead and prescribe. Will you tell me what kind of situations that this might occur?
    b. Will you share with me your personal experience in this type of situation?

2. We talked about before how providers may prescribe antibiotics even though there may be reasons for them not to. I'm wondering the same question for NSAIDs, why might a provider prescribe an NSAID even though there may be reasons for them not to?
    a. Will you share with me your personal experience in this type of situation?

3. What challenges arise when communicating with patients about these medications?
    c. Probe: If the patient has an expectation (antibiotics, NSAIDs) that is not clinically appropriate, how do you help the patient understand? How does that discussion go?

II. <u>Knowledge and Awareness</u>

1. In your current practice, walk me through the decision-making process for how you make prescription decisions? Do you ever consult guidelines, ED pharmacist, colleagues in real-time?
2. What other resources that would be useful?
3. At your site, do you think that antibiotics are prescribed when maybe they shouldn't be?
    a. Why do you think this occurs?
    b. What about NSAIDs?
    c. Why do you think this occurs with NSAIDs?
4. Do you ever hear about adverse events when these medications are prescribed?
    a. What's your experience with that?
5. What existing efforts outside of your control (e.g., hospital policies, ongoing quality improvement programs, EHR (Electronic Health Record) tools, pharmacist) influence how you prescribe these medications? Are there any other tools or people who help? Probe: Why/how?
    a. What about Antibiotics vs. NSAIDs?
6. If you found out that an antibiotic or NSAID caused harm to one of your patients, how would this influence your prescribing?
7. If one of these agents caused harm to one of your colleagues' patients, do you think this knowledge would influence your practice?

a. There are several interventions to facilitate change, which would you find most helpful? Why? (go through each individually- how and why useful/not useful)

- Learning about your own prescribing patterns;
- Specific patient adverse events;
- Real-time clinical decision support? Leadership support?
- Peer expert (e.g., pharmacist) providing counseling;
- Incentives to follow-up on patients (e.g., CME, maintenance of certification).
- Anything else that would be useful?

III. Integration into Practice: We are planning an intervention focused on prescribing of antibiotics and NSAIDs in VA unscheduled care settings (ED, Urgent Care Clinics, primary care). The following questions we will discuss pertain to this intervention:

1. Please review the following sample feedback report (show wireframe and give brief orientation), and tell us your thoughts about this draft.
    1. Would you use this?
    2. Taking this as an example report, what characteristics about such a report would make you most willing to review this?
    3. What additional content would be useful to you?
    4. Which content can be removed?
    5. Differ by NSAID vs. antibiotic? Should this be tailored differently for NSAIDs vs. Antibiotics?
    6. Differ by setting? (ED, Urgent Care Clinics, Primary Care)
    7. How would you like to receive the report (e.g., email, text, paper report)?
    8. When/how often would you like to receive this?
    9. What else would make this useful/even better?
2. If you were provided a feedback report similar to this, what would you do with these data?
    1. Is there anything that would make you want to use these data more?
    2. What are some ways to get providers to use and think about the report?
    3. Would you be more willing to review the report if you were offered CME, MOC, or credit for quality improvement (as part of your performance bonus) in exchange?
    4. Any other incentives or ideas?
3. If a pharmacist were to approach you to discuss your prescribing, in what way would you like them to do this?

Conclusion

1. This has been so helpful. Again, as a non-clinician, is there anything that I didn't ask that I should have? (something to this effect- I have found this useful to extract more information/opinion) Is there anything else you would like us to know in regard to prescribing of antibiotics and NSAIDs?

C. Stakeholder Interview Guide

Introductory Questions

1. Please describe your general responsibilities in your role(s).

The ultimate goal of our research program is to improve the quality of prescribing of antibiotics and non-steroidal anti-inflammatory drugs (NSAIDs, e.g., ibuprofen) in acute care settings in the VA (emergency department, urgent care clinic, and primary urgent care). We are planning an intervention focused on prescribing of antibiotics and NSAIDs at the Nashville and Murfreesboro VA for all acute outpatient care providers that includes academic detailing, an automated prescribing feedback system, and non-financial incentives (e.g., continuing medical education and/or maintenance of certification).

Key Questions

I. Determinants of Potentially Inappropriate Prescribing

1. What do you think are the main reasons medications are prescribed when perhaps they should not be?
    a. Prompt: Clinician awareness, knowledge, beliefs, patient communication
2. What are some of the reasons that could be unique to antibiotic prescription? What are some of the reasons that could be unique to NSAIDs?
3. (Describe project again/goal): What, if any, experience do you have in dealing with prescriptions in ED/UCC/unscheduled primary care settings (e.g., prescribing, supervisory etc)?
4. What do you think will be our main barriers in addressing potentially inappropriate prescribing?
    a. Potential examples: Competing priorities, too many feedback reports, provider buy-in
    b. How can we mitigate this in our planned intervention?
5. What do you think are some possible strategies we can use to address this problem?
    a. How could we apply those?

II. Integration into Practice

1. (Give overview of wireframe) Please review the following sample feedback report (show wireframe), tell me what you think about it.
    a. What about this do you think would be unclear to a user? Or not intuitive?
    b. What would be the single most important measure we should provide to clinicians reviewing this?
    c. Probe: How should this be tailored differently for NSAIDs vs. Antibiotics?
    d. What is the most meaningful way to deliver the report to a provider? How should the report be received (e.g., email, text, paper report, in-person)?
2. When/how often should a provider receive a report like this? What should providers do with these data?
    a. Probe: Engagement, reflection?
    b. Should Supervisors have access?
3. Are you aware of any existing reports that already do this?
    a. Limitations? Alternative resources? Groups?
4. Do you think the use of continuing medical education (CME) and/or maintenance of certification (MOC) credits, and/or counting towards quality metric for annual performance pay would motivate a provider to review the report?
    a. Alternative incentives?
5. On a national scale, how should this intervention be implemented in clinical practice?
6. We would like to eventually implement this intervention on a broader (e.g., regional or national level), what potential barriers do you think we will face?

a. How to overcome?

7. What are some potential facilitators/strategies?

III. **Conclusion**

1. Are there other individuals or groups we should speak to about this intervention?

CRAFT – Prescribing Feedback Coding System

© 2023

SUPPLEMENTAL TABLE 2. Coding Framework

Category	Label	Definition	Rules and notes
**1**	**Inner Setting**	**Participant discusses inner setting characteristics of her/his clinical practice**	**CFIR Constructs**
1.1	Culture of clinic/organization	Norms, values, and basic assumptions of a given organization, unique aspect of clinic (e.g., patient population; patient-centered; competitive)	
1.2	Implementation Climate	The absorptive capacity for change, shared receptivity of involved individuals to an intervention, and the extent to which use of that intervention will be rewarded, supported, and expected within their organization.	
1.2.1	Tension for Change	The degree to which stakeholders perceive the current situation as intolerable or needing change.	
1.2.2	Compatibility	The degree of tangible fit between meaning and values attached to the intervention by involved individuals, how those align with individuals' own norms, values, and perceived risks and needs, and how the intervention fits with existing workflows and systems.	
1.2.3	Relative Priority	Individuals' shared perception of the importance of the implementation within the organization.	
1.2.4	Organizational Incentives & Rewards	Extrinsic incentives such as goal-sharing awards, performance reviews, promotions, and raises in salary, and less tangible incentives such as increased stature or respect.	
1.2.5	Goals and Feedback	The degree to which goals are clearly communicated, acted upon, and fed back to staff, and alignment of that feedback with goals.	
1.2.6	Learning Climate	A climate in which: a) leaders express their own fallibility and need for team members' assistance and input; b) team members feel that they are essential, valued, and knowledgeable partners in the change process; c) individuals feel psychologically safe to try new methods; and d) there is sufficient time and space for reflective thinking and evaluation.	

1.3	Organizational readiness for Implementation	Tangible and immediate indicators of organizational commitment to implement an intervention.	
1.3.1	Leadership Engagement/commitment	Commitment, involvement, and accountability of leaders and managers with the implementation.	
1.3.2	Available Resources	The level of resources dedicated for implementation and on-going operations, including money, training, education, physical space, and time are identified and available.	
1.3.3	Access to Knowledge & Information	Ease of access to digestible information and knowledge about the intervention and how to incorporate it into work tasks.	
1.4	Other inner setting discussion	Other issues related to inner setting not listed above	
**2**	**Networks and Communication**	**Discussion centering on the quality of communication**	
2.1	Between providers	Discussion centers on communication between providers	
2.2	Interprofessional engagement	Knowledge of interprofessional roles and responsibilities (e.g., can consult pharmacist if have question, consult with allied health)	
2.3	Between patients and providers	Discussion focuses on the communication that occurs between patients and providers	
2.4	Family	Discussion focuses on communication with patient's family	
2.5	Other communication	Other communication not listed above	
2.6	Communication Modalities	Discussion centers on mode of communication	
2.6.1	In-person	Discussion centers on in-person communication	
2.6.2	EMR basket/note	Discussion centers on provider communication by way of EMR, notes, etc.	Communication between interprofessional team
2.6.3	Phone	Discussion between providers via phone	Do not code for patient communication
2.6.4	Telehealth (i.e. phone, video, portal)	Discussion centers on patient communication through telehealth	
2.6.5	Other communication modality	Discussion centers on other forms of communication	
**3**	**Outer Setting**	**Participant discusses outer setting characteristics**	**CFIR Constructs**

3.1	Patient Needs & Resources	The extent to which patient needs, as well as barriers and facilitators to meet those needs, are accurately known and prioritized by the organization. (e.g., social determinants/disparities; patient-centered care)	
3.2	Cosmopolitanism	The degree to which an organization is networked with other external organizations.	
3.3	Peer pressure/competing organizations	Mimetic or competitive pressure to implement an intervention; (e.g., key peer or competing organizations have already implemented or are in a bid for a competitive edge.)	
3.4	External Policy & Incentives	A broad construct that includes external strategies to spread interventions, including policy and regulations (governmental or other central entity), external mandates, recommendations and guidelines, pay-for-performance, collaboratives, and public or benchmark reporting.	
3.5	Ongoing projects/changes	Existing activities beyond control, e.g. ongoing QI projects	
3.5.1	Specific dashboard experience	(e.g. Opioid dashboard, antimicrobial stewardship dashboard)	
3.6	Clinical Informatics/Health Information Technology	Discussion centers on CPRS, IT, systems, etc.)	
3.7	Knowledge of protocols and processes	Knowledge and understanding of clinic-specific requirements or restrictions (e.g., VA only allows a list of meds they can use)	If attribution is being made, also code with category 6.1.2
3.8	Support decision tools/guidelines	Knowledge/utilization of guidelines for prescription decisions, use clinical decision tools, etc.	
**4**	**Patients-level influence/factors**	**Patient-level factors that influence or impact prescribing decision**	**CFIR construct outer setting; will often co-occur with code 3.1**
4.1	Expectation	Patient expectation of prescription or other outcome, etc.	
4.2	Compliance with physician decision	Discussion centers on patient's agreement/acceptance of physician decision	
4.3	Condition/symptoms	Discussion centers on patient's condition and symptoms	Code overall 4.3 if referring to general symptoms; Code this for patient response to reason for visit.
4.3.1	Pain	Patient presents with pain	
4.3.2	Infection	Patient presents with potential infection	
4.3.3	Diagnosis	Discusses formal diagnosis (e.g., UTI, cold)	

4.3.4	Other condition/symptom	Other condition not listed above	
4.4	Magnitude of symptoms	Discussion centers on intensity and/or trajectory of patient disease	
4.5	Adverse events/risks	Discussion centers on patient adverse events (requires intervention)-experienced or potential risk	
4.6	Comorbidities	Discussion centers on patient's comorbid conditions	
4.7	Social/economic/logistical	Patient factors such as socioeconomic status; environment, resources	
4.8	Age	Discussion includes patient's age	
4.9	Patient follow-up	Discusses patient follow-up (e.g., "if I am aware of an issue" question in interview guide)	
4.10	Habits	Patient habits, such as smoking	Can include useful habits (e.g., exercise)
4.11	Group comparison	Discussion includes patient comparison (e.g., gender, age)	
4.12	Patient Education	Discussion centers on patient education techniques	
4.12.1	Information needs	Discussion centers on patient information needs regarding NSAIDS, antibiotics, or other treatments	
4.12.2	Opinion of mode of education	(e.g. Discussion with provider, in person one on one, Handouts/ pamphlets, Websites, Posters in the room, MyHealththeVet message portal)	
4.12.3	Patient centered	Patient-centered care such as avoiding jargon, shared decision making, etc.	
4.12.4	Sources of information (what patients currently seek on own)	Information sources that veterans seek (e.g., online, friends, family, etc.)	
4.12.5	Drug Interactions	When Veteran asks questions about other medications they are on	
4.12.6	Dosage	When Veteran asks questions about medication dosage	
4.12.7	Other patient education	Other patient education discussion not listed above	
4.13	Side Effects	(e.g. Weight gain, nausea, vomiting, diarrhea, etc.)	

4.14	Allergy	(e.g. Allergic reaction, allergic to medication, etc.)	
4.15	Other patient factor	Other patient factor not listed above	
4.16	Attitudes and beliefs	Veteran code (e.g. thoughts, beliefs)	
4.17	Preferences	Any discussion of patient preferences (e.g., facility location, medication, etc.)	
4.18	Emotions	Veteran emotions such as trust, anger, etc.	
**5**	**Intervention characteristics**	**Discussion centers on characteristics of the proposed intervention**	
5.1	Wireframe presentation	Discussion focuses on the existing wireframe prototype	
5.1.1	Peer comparison section	Participant provides feedback on peer comparison section of wireframe	
5.1.2	Notable events history	Participant provides feedback on notable events history section of wireframe (provider events)	
5.1.3	Patient notable events	Participant provides feedback on patient notable events section of wireframe	
5.1.4	Tips box	Participant provides feedback on Tips section of wireframe	
5.1.5	Sidebar	Participant provides feedback on the sidebar of wireframe	
5.1.6	Other wireframe discussion	Other wireframe discussion not listed above	
5.2	Design Quality & Packaging	Perceived excellence in how the intervention is bundled, presented, and assembled.	
5.2.1	Design Layout	Comments on the design or layout of the sample wireframe	
5.2.2	Content/data	Comments on the content/data of the sample wireframe	
5.2.3	Experience with similar programs	Discussion centers around an experience that the clinic has had with a similar program(s)	
5.2.4	Other design characteristic	Other intervention characteristics not listed above	
5.3	Selected strategies	Specific intervention component or strategy	

5.3.1	Audit and Feedback	Use of EMR data to generate visual summaries as feedback	Related to dashboard and the feedback that providers will obtain from it
5.3.2	Academic Detailing	Meetings with a colleague to receive feedback (Pharmacist meeting to review the provider's dashboard	
5.3.3	Incentive strategy	Discusses rewards for meeting goals (e.g., CME, MOC, QI credit)	
5.3.4	Tool development	Methods used to create and develop the informatics tool/dashboard	
5.4	Intervention Source	Perception of key stakeholders about whether the intervention is externally or internally developed	
5.5	Evidence Strength & Quality (outcome specific)	Stakeholders' perceptions of the quality and validity of evidence supporting the belief that the intervention will have desired outcomes.	
5.6	Relative Advantage	Stakeholders' perception of the advantage of implementing the intervention versus an alternative solution.	
5.7	Adaptability	The degree to which an intervention can be adapted, tailored, refined, or reinvented to meet local needs.	
5.8	Trialability	The ability to test the intervention on a small scale in the organization, and to be able to reverse course (undo implementation) if warranted.	
5.9	Complexity	Perceived difficulty of implementation, reflected by duration, scope, radicalness, disruptiveness, centrality, and intricacy and number of steps required to implement.	
5.10	Cost	Costs of the intervention and costs associated with implementing the intervention including investment, supply, and opportunity costs.	
5.11	Potential source of confusion	Participant asks a clarifying question or expresses confusion about an intervention characteristic	
**6**	**Individual characteristics**	**Constructs related to beliefs, motivation, behaviors of individuals**	
6.1	Behavioral Beliefs	Provider knowledge of problem, guidelines, adverse outcomes related to NSAID or Antibiotic prescription; (impacts/outcomes of behavior change)	
6.1.1	Knowledge	Provider knowledge of problem, guidelines, adverse outcomes related to NSAID and/or antibiotic prescription	

6.1.1.1	Aware of prescribing problem	Provider is aware of problems associated with prescribing patterns	
6.1.1.2	Unaware of prescribing problem	Provider is unaware or has limited awareness of problems associated with prescribing patterns	
6.1.2	Causal attributions related to problem	Discussion centers on causal attributions related to prescribing practices	
6.1.2.1	Internal causal attribution	Attributions of her/his own thoughts or abilities, elements of personality	
6.1.2.2	External causal attribution	Situational attributions unrelated to her/his actions	
6.1.3	Magnitude of problem	Perceived magnitude of the problem with over prescription	
6.2	Normative Beliefs	Attitudes and values placed on the intervention; familiarity of facts associated with intervention.	
6.2.1	Intervention-specific normative beliefs	Participant's attitudes toward and value placed on the intervention as well as familiarity with facts, truths, and principles related to the intervention	
6.2.1.1	Familiarity with facts	Familiarity with facts, truths, and principles related to intervention	
6.2.1.2	Value placed on the intervention	Attitudes and value associated with intervention	Also code if perception that others would use the intervention
6.2.2	Organizational culture-specific normative beliefs	Perceptions of behaviors and expectations associated with the problem and associated with the intervention	
6.2.2.1	Descriptive organizational beliefs	Perception of the behavior of others or behaviors that are typically performed within the organization	
6.2.2.2	Injunctive organizational beliefs	Perceptions of behavior that s/he is expected or will be expected to perform	
6.2.2.3	Anticipated outcomes	Anticipated outcomes related to a given behavior or intervention	
6.3	Control Beliefs	Factors that facilitate or impede performance of a given behavior; her/his perceived ability to influence the implementation or other health system outcomes	

6.3.1	Self efficacy/perceived behavioral control	Confidence or beliefs in one's ability to perform a task or utilize the intervention	
6.3.2	Individual Stage of Change	Characterization of the phase an individual is in (e.g., enthusiastic about the intervention)	
6.3.3	Other control beliefs	Other beliefs about control not listed above	
6.4	Most helpful aspects (would this be useful)	Interview guide question, "There are several interventions to facilitate change, which would you find most helpful? Why? (go through each individually- how and why useful/not useful)	Will co-occur with other codes- this is for a designated question
6.5	Data delivery preferences	Discussion centers on preferences related to intervention data delivery	
6.5.1	Mode of delivery code	Discussion centers on mode of data delivery (e.g., email, paper report)	
6.5.2	Frequency of delivery	Discussion centers on how often on should receive these data	
6.5.3	Timing	Discussion centers on time of day data should be received	
6.6	Likes/useful	Specific aspects of the intervention that the participant expresses to like or find useful	
6.7	Dislikes/not useful	Specific aspects of the intervention that the participant expresses to dislike or does not find useful	
6.8	Concerns	Expresses specific concern about intervention	
6.9	Current practice	Discussion centers on her/his current decision-making process related to prescription	Code if specifying her/his current behaviors
6.10	Stakeholder compare/contrast	Stakeholders comparison (e.g. providers, settings, locations)	Code for stakeholder interviews only
**7**	**Implementation Process and considerations**	**Discussion centers on aspects of intervention that should be considered for implementation**	
7.1	Engaging (strategy specific)	Attracting and involving appropriate individuals in the implementation and use of the intervention through a combined strategy of social marketing, education, role modeling, training, and other similar activities.	CFIR process
7.2	Leadership engagement and commitment	Commitment, involvement, and accountability of leaders and managers with the implementation.	CFIR inner setting

7.3	Opinion Leaders/person of influence	Individuals in an organization who have formal or informal influence on the attitudes and beliefs of their colleagues with respect to implementing the intervention.	CFIR process
7.4	Formally Appointed Internal Implementation Leaders	Individuals from within the organization who have been formally appointed with responsibility for implementing an intervention as coordinator, project manager, team leader, or other similar role. (ex. Champion)	
7.5	External Policy & Incentives	A broad construct that includes external strategies to spread interventions, including policy and regulations (governmental or other central entity), external mandates, recommendations, CME, MOC, credit for quality improvement (as part of your performance bonus)	CFIR outer setting
7.6	Reflecting & Evaluating	Discussion centers on utilizing the data to reflect on her/his practice and make changes.	
7.7	Clarifying question	about implementation (participant not necessarily confused)	
**8**	**Behavioral Intentions**	**Discussion about how they may change their behavior in the future**	
8.1	Intention to change	The participants is clear that they intend to change a behavior	
8.2	Intention to consider behavior change	The participant will consider, contemplate, or evaluate whether or not to change a behavior	
8.3	No intention to change	The participant states clearly that the intention is to NOT change behavior	
8.4	Prescribing behavior intentions	Behaviors to be implemented antibiotics or NSAID	
8.4.1	NSAID intention	Changes in prescribing behaviors for NSAIDS	
8.4.2	Antibiotic intention	Changes in prescribing behaviors for antibiotics	
8.4.3	Other therapeutic changes	Other behavior changes in practice	Co-occur with other therapeutics except for NSAIDS and antibiotics
8.5	Stakeholder-specific comment	Stakeholder specific comment about behavior (thoughts, beliefs)	When stakeholders specifically discuss their own beliefs/behavioral intentions of providers or clinics
**9**	**Therapeutics**	**Discussion is specific to type of medication**	

9.1	Pharmaceuticals	Discussion centers on pharmaceutical interventions	
9.1.1	NSAIDS	(e.g. Aspirin, Ibuprofen, Naproxen)	Likely not co-occur with 8.4 categories
9.1.2	Antibiotics	(e.g., Amoxicillin, Clindamycin, etc.)	Likely not co-occur with 8.4 categories
9.1.3	Opioid	(e.g. Oxycodone, morphine, etc.)	
9.1.4	Alternatives	(e.g., Tylenol, over the counter medications)	
9.1.5	Complimentary care	Non drug and surgical interventions (ex. Mindfulness, yoga, psychotherapy)	
9.1.6	Physical Therapy	Physical Therapy	
9.1.7	Characterization of treatments	Treatment outcomes such as impact on health, medication reconciliation, etc.	
9.1.8	Other therapeutic	Other types of therapies not listed above	
**10**	**Barriers/Facilitators**	**Participant describes a potential challenges or facilitators**	**Will co-occur with other codes**
10.1	Barriers	Discussion centers around a barrier or disadvantage	
10.2	Facilitator	Discussion centers around a facilitator or advantage	
10.3	Contingency/conditional	Decision or choices depends upon specified factors	
**11**	**Suggestions and solutions**	**Suggestions provided by participant**	
11.1	Additional feature	Suggestion for additional intervention feature	
11.2	Remove feature	Suggests to remove a given intervention feature	
11.3	Modification	Modification of intervention feature	
**12**	**Example**	**Provides an example experiences related to prescribing or hypothetical situations**	

12.1	Went well	Example situations that went well	
12.2	Not go well	Example situations that did not go well	
12.3	General description	General example not specified as went well or did not go well	
12.4	Hypothetical example	Provides hypothetical example	
**13**	**Settings of care**	**Discussion centers on setting of care**	
13.1	ED	Emergency department setting	
13.2	Urgent care	Urgent Care setting	
13.3	Outpatient	Outpatient setting	
13.4	Supervisory	Supervisory setting	
13.5	Prescription	Prescription setting- use for stakeholder	
13.6	Inpatient	Inpatient setting	
13.7	Primary Care	Primary care setting	
13.8	Other setting	Other setting not listed above	
**14**	**World events**	**World events such as Covid-19, opioid epideictic, etc.**	
**15**	**Not Observed**	**Participant has not observed/experienced a given event**	
**16**	**Change over time**	**Any discussion related to change over time**	
**17**	**Participant details**	**Participant describes her/his background, demographics, roles and responsibilities, etc.**	**There will be a designated column for these data in the coding sheet.**
17.1	Age	Participants age in years	
17.2	Gender	Participants gender	
17.2.1	Female	Participant identifies as female	

17.2.2	Male	Participant identifies as male	
17.3	Clinical setting	Clinical environments in which s/he works	
17.3.1	Outpatient	Participant works in Primary care setting	
17.3.2	Urgent care	Participant works in Urgent care setting	
17.3.3	ED	Participant works in Emergency Department Setting	
17.3.4	Other clinical setting	Other clinical setting not listed above	
17.4	Years of experience	Participants years of experience (in years only)	
17.5	Role	Participant's role in clinical Setting	Also code if word unit mentions her/his role (e.g., "as a PCP I am able to…)
17.5.1	Nurse	Participant is a Nurse	
17.5.2	Pharmacist	Participant is a Pharmacist	
17.5.3	Advanced practice provider	Participant is an Advanced Practice Provider	
17.5.4	Physician	Participant is a Physician	
17.5.5	Clinical leader	Participant is a clinical leader	
17.5.6	Operational/administrative leader	Participant is an operational or administrative leader	
17.5.7	Other role	Participant has other role not listed above	
17.6	Patients per shift	Number of patients participant sees per shift (If gives range, go with highest number)	
17.7	Responsibilities	Participant describes her/his responsibilities	
17.7.1	Patient care	(e.g. Responsibilities related to Emergency Department)	
17.7.2	Administrative/documentation	(e.g. Responsibilities related to administrative work or documentation)	
17.7.3	Other responsibilities	Other responsibility not listed above	

17.8	How often for infection	Describes how often s/he sees patients for infection	This will be entered into coding sheet in text format
17.9	How often for pain	Describes how often s/he sees patients for pain	This will be entered into coding sheet in text format
17.10	Other Participant Characteristics	Other participant characteristics not listed above	
**18**	**Exemplary Quotes**	**Notable quotes that express data most effectively**	

SUPPLEMENTAL TABLE 3. Major Coding Categories for Qualitative Analysis

1) Inner setting;<br>2) Networks and communication;<br>3) Outer setting;<br>4) Patient-level influences/factors;<br>5) Intervention characteristics;<br>6) Individual characteristics;<br>7) Implementation process and considerations;<br>8) Behavioral intentions;<br>9) Therapeutics;<br>10) Barriers and facilitators;<br>11) Suggestions and solutions;<br>12) Example experiences related to prescribing;<br>13) Settings of care;<br>14) World events;<br>15) Not observed;<br>16) Change over time;<br>17) Participant details;<br>18) Exemplary quotes.
